# PEAK Mood, Mind, and Marks: a pilot study of an intervention to support university students’ mental and cognitive health through physical exercise

**DOI:** 10.3389/fpsyt.2024.1379396

**Published:** 2024-06-10

**Authors:** Catherine E. B. Brown, Karyn Richardson, Bengianni Halil-Pizzirani, Sam Hughes, Lou Atkins, Joseph Pitt, Murat Yücel, Rebecca A. Segrave

**Affiliations:** ^1^BrainPark, Turner Institute for Brain and Mental Health, Monash University, Melbourne, VIC, Australia; ^2^Centre for Behaviour Change, University College London, London, United Kingdom; ^3^Queensland Institute of Medical Research (QIMR) Berghofer Medical Research Institute, Brisbane, QLD, Australia

**Keywords:** university students, physical exercise, mental health, cognitive health, brain health, behavioural intervention, implementation evaluation, COM-B

## Abstract

**Introduction:**

Regular exercise has the potential to enhance university students’ mental and cognitive health. The PEAK Mood, Mind and Marks program (i.e., PEAK) is a neuroscience-informed intervention developed using the Behaviour Change Wheel to support students to exercise three or more times per week to benefit their mental and cognitive health. This pilot study assessed the impact of PEAK on exercise, mental and cognitive health, and implementation outcomes.

**Methods:**

PEAK was delivered to 115 undergraduate university students throughout a 12-week university semester. The primary outcome was weekly exercise frequency. Secondary outcomes were: time spent engaged in moderate-vigorous exercise, sedentary behaviour and perceived mental health and cognitive health. All were measured via online self-report questionnaires. Qualitative interviews with 15 students investigated influences on engagement, the acceptability and appropriateness of PEAK, and its mechanisms of behaviour change. Paired t-tests, Wilcoxon Signed-Rank tests and template analysis were used to analyse quantitative and qualitative data, respectively.

**Results:**

On average, 48.4% of students engaged in the recommended frequency of three or more exercise sessions per week. This proportion decreased towards the end of PEAK. Sedentary behaviour significantly decreased from baseline to end-point, and moderate-vigorous exercise significantly increased among students’ who were non-exercisers. Mental wellbeing, stress, loneliness, and sense of belonging to the university significantly improved. There were no significant changes in psychological distress. Concentration, memory, and productivity significantly improved. Sixty-eight percent of students remained engaged in one or more components of PEAK at end-point. Qualitative data indicated students found PEAK to be acceptable and appropriate, and that it improved aspects of their capability, opportunity, and motivation to exercise.

**Conclusions:**

Students are receptive to an exercise-based program to support their mental and cognitive health. Students exercise frequency decreased; however, these figures are likely a conservative estimate of students exercise engagement. Students valued the neuroscience-informed approach to motivational and educational content and that the program’s goals aligned with their academic goals. Students identified numerous areas PEAK’s content and implementation can be optimised, including use of a single digital delivery platform, more opportunities to connect with peers and to expand the content’s cultural inclusivity.

## 1 Introduction

The mental and cognitive health of university students play a pivotal role in their academic success and post-university career prospects. However, a large proportion of students experience difficulties with these aspects of health. Globally, 35% of university students meet criteria for one or more mental illnesses (1), 65% report high levels of psychological distress (2), and 36% report feeling lonely (3). Concerns about cognitive performance are similarly common, with 60% describing their cognitive capacity as sub-optimal in domains including memory and concentration (4). Addressing students’ mental and cognitive health challenges is important for supporting students’ broader quality of life and academic performance.

Promoting regular physical exercise^1^ has the potential to be an effective and empowering approach to address these concerns. A robust body of evidence demonstrates that regular exercise has substantial positive impacts on mental and cognitive health. For example, regular exercise, especially when performed at moderate to vigorous intensities, can be as effective as psychotherapy and pharmacotherapy in reducing symptoms of depression and anxiety (6). Beneficial mental health effects have also been established in other stress-related disorders, schizophrenia, and substance-use disorders (7–12). Exercise is also associated with powerful reductions in the overall burden of poor mental health. For example, a study involving 1.2 million adults (13) demonstrated that people who were physically active in the preceding month had 43% fewer days of poor mental health during the month compared to those that were not active. Interestingly the benefits were non-linear, with greatest benefits associated with exercising three to five times per week for 30 - 60 minutes per session. These findings are consistent with a large body of longitudinal research demonstrating that engaging in exercise regularly is associated with improved mental health among a range of clinical and non-clinical populations (14), including university students (15). Specifically, engaging in regular exercise can buffer the negative effects of stress on mental and physical health (16, 17), enhance psychological wellbeing (18, 19), reduce psychological distress (20) and, especially when performed with other people, can reduce social isolation and loneliness (21).

A large and growing body of evidence has also demonstrated that exercise confers significant cognitive health benefits. Cognitive health benefits encompass improvements in memory, heightened efficiency of attentional processes, and strengthened executive-control processes (22–26). Within this literature, the impacts of different types of exercise have been evaluated, with most attention focused on aerobic exercise and, increasingly, resistance training. Regular aerobic exercise can induce improvements in memory and executive functioning skills (27, 28), including working memory, cognitive flexibility, and inhibitory control (29). Similarly, improvements in executive functioning skills have been demonstrated following regular resistance training (30), such as selective attention and inhibitory control (31, 32). While studies investigating the cognitive benefits of exercise have tended to focus on older adults, prospective studies have reported positive associations between exercise and cognitive function among young adults (33), suggesting that higher levels of exercise may help to maintain cognitive health throughout adulthood.

A key mechanism by which engaging in exercise is thought to promote mental and cognitive health is via its impacts on neuroplasticity (34–39). When performed regularly and at moderate and vigorous intensities, exercise can have profound effects on the structure, connectivity, and functioning of the brain (34, 40, 41). While the molecular mechanisms of exercise-induced neuroplasticity are only partially understood, one key pathway that has been extensively studied is the impact of exercise intensity on brain-derived neurotrophic factor (BDNF) (42, 43), a key signalling molecule involved in neuroplastic changes related to learning and memory (44). During vigorous intensity exercise, the demand for energy rapidly escalates, which triggers a series of processes that increases circulating lactate (45, 46). Circulating lactate can pass through the blood brain barrier and, when it does so, catalyses an increase in BDNF (47). A second neuroplastic mechanism is the impact of exercise on insulin-like growth factor 1 (IGF-1) (48). For example, when engaging in resistance exercise, Human Growth Hormone is secreted from the pituitary gland, which promotes the release of IGF-1 from the liver into the bloodstream (49, 50). IGF-1 can, in turn, cross the blood brain barrier (51, 52). Once in the brain, IGF-1 plays a critical role in brain development and cognition and, at low levels, is a marker of cognitive decline and dementia (53). While numerous activities (e.g., aerobic training, a high protein diet, sleep) can also lead to the release of IGF-1 (54–57), strength training has been shown to be particularly powerful at eliciting IGF-1 release. Exercise-induced neuroplastic changes have been linked to improvements in mental and cognitive health outcomes, including depression (58, 59), psychological wellbeing (19), episodic and verbal memory (60, 61), and global cognitive performance (62). Educating students about these neuroscientific mechanisms could enhance students’ engagement in an exercise-based intervention for the benefit of their mental and cognitive health (63). While knowledge alone is not sufficient to change behaviour, it is a necessary component and can increase the acceptability of an intervention (64, 65).

An exercise-based intervention may be appealing to students if it aligns with their academic goals. Students understandably tend to prioritise performing well at university over initiating or maintaining a regular exercise routine (66). Therefore, programs that emphasise the positive brain effects of exercise and neuroscientific evidence that demonstrates exercise can optimise cognition and buffer stress to support academic performance could be particularly engaging (63). Offering students’ a program to support their mental and cognitive health may also help universities to achieve strategic priorities, including attracting and retaining students, by strengthening students’ sense of belonging to the university (67, 68).

The PEAK Mood, Mind, and Marks program (i.e., PEAK) is a neuroscience-informed behaviour change program designed to increase university students’ engagement in exercise (63). PEAK was developed using the behaviour change wheel (BCW), a gold-standard behavioural intervention development framework (69), and heavily informed by co-design consultations with students, staff, and senior leadership (63). The Capability, Opportunity, Motivation, Behaviour (COM-B) model is at the centre of the BCW and posits that changing behaviour involves targeting one or more of a person’s capability, opportunity, and motivation. Within these three components, capability can be psychological (e.g., knowledge to engage in the necessary processes) or physical (e.g., physical skills); opportunity can be social (e.g., interpersonal influences) or physical (e.g., environmental resources); and motivation can be automatic (e.g., emotional reactions, habits) or reflective (e.g., conscious plans, intentions, beliefs). The PEAK program content and messaging were designed to target students’ barriers and facilitators to behaviour change, including their goals to excel academically. This approach distinguishes PEAK from other health behaviour change interventions for university students, testing a novel strategy to support students to exercise regularly for the benefit of their ‘mood, mind, and marks’.

Best practice for developing behavioural interventions like PEAK includes multiple rounds of small-scale piloting and iteration before large scale evaluation and roll out (69–72). This kind of early iterative testing provides critical insights that can be used to refine the program design to optimise the acceptability, feasibility, usability, and efficacy of the program. Evaluation of behavioural and health impacts and implementation outcomes is useful to inform how well the program is meeting the needs of the students who take part, and how the program can be improved. Thus, the aim of the present study was to conduct a pilot of the PEAK program and: (i) evaluate its impacts on students’ exercise engagement and perceived mental and cognitive health, and (ii) to assess implementation outcomes across student engagement, barriers and enablers to engagement, the acceptability and appropriateness of PEAK, and mechanisms of behaviour change.

## 2 Materials and methods

### 2.1 Study design

A quasi-experimental single-arm pilot study design was adopted and the simultaneous assessment of program impact and implementation outcomes (see (73)) was conducted. This design is especially pertinent for evaluating programs in the tertiary education system because such a design can be used to evaluate the success of implementation strategies in the engagement and uptake of PEAK at the individual-level. The program was piloted throughout July – November 2022, coincident with the second semester of an Australian university teaching period. The Template for Intervention Description and Replication (TIDieR) checklist is provided in **Supplementary File 1**.

### 2.2 Participants

Students were eligible if they were aged 18 years or over, currently enrolled in an undergraduate degree at Monash University (Melbourne, Australia), and able to provide voluntary informed consent. Exclusion criteria included self-reported contraindications to safe engagement in regular exercise as identified by the Physical Activity Readiness Questionnaire (PAR-Q) (74) (e.g., a heart condition, high blood pressure, or chest pain while participating in physical activity) or lifetime diagnosis of an eating disorder.

Stratified random sampling was used to enrol students based on whether they were domestic or international students, ensuring the representation across these two groups was approximately equal to assess the suitability and impact of PEAK for both cohorts. Recruitment was conducted throughout May – July 2023 via advertisements placed on-campus and student social media pages. Eligibility for participation was determined via a brief online screening questionnaire. All participants provided informed consent online and the study was approved by the Monash University Human Research Ethics Committee (#32395).

### 2.3 Intervention

For a detailed description of the intervention co-design process, program content, and delivery methods see Brown et al. (in review). In brief, PEAK is a 12-week semester-long program focused on supporting students to engage in three or more sessions of exercise per week, with a session understood to be 10-minutes or more of exercise. PEAK was designed to target students’ capability, opportunity, and motivation to exercise (see (66) for a systematic review) by implementing eight core program components (see **Table 1** for a description of each component). Program components were delivered via digital channels (i.e., Moodle, WhatsApp, Qualtrics, text message, email) and a small number of in-person activities at Monash University. A stratified sample of domestic and international students participated in the co-design and evaluation of PEAK, offering a diversity of perspectives to develop an inclusive program.

**Table 1:** PEAK program components, mode of delivery and delivery schedule.

Program component	Description	Mode of delivery	Delivery schedule
“Kick Off session” PEAK program onboarding	Students were invited to attend a “Kick Off Session” to learn about PEAK’s purpose, how to participate, and how exercise can improve mental and cognitive health. Students had the opportunity to meet peer participants and the PEAK program team at the session.	In person	Baseline
Menu of exercise options and goal	Students were informed that the core PEAK program exercise goal is to build up to exercising three times per week for 12-weeks.	Digitally via Moodle and in person	Baseline
	Students were given an “exercise starter kit” which included an exercise mat, drink bottle and towel to enable online exercise options at home, a calendar to schedule their exercise sessions, and 12 free passes to access the university gym for gym use and/or group exercise sessions on-campus.	In person	Baseline
	Each week, students were given access to a new menu of free exercise options curated by a senior exercise physiologist that comprised: aerobic and resistance training options; beginner and advanced options; digital and in-person training options; options that did not require exercise equipment.	Digitally via Moodle	Week 1 - 12
Twelve weekly educational and motivational videos*	Each week, students received a new educational or motivational video designed to target common barriers to exercise most often experienced by university students. Core messages reiterated across these videos included: how to build and sustain a regular exercise routine; how to overcome common student’ barriers to exercise; how the mental and cognitive health impacts of regular exercise can support academic performance; the key neuroscientific mechanisms through which vigorous intensity exercise and resistance exercise can change the structure and function of the brain to support mental and cognitive health.	Digitally via Moodle	Week 1 - 12
“PEAK Points” reward system	Students received a notification that they were eligible to earn one ‘PEAK Point’ for each completed exercise session. Points could be redeemed for coffee vouchers for the student union on-campus café or small group training sessions led by the PEAK team exercise physiologist.	Digitally via text message	Week 1 - 12
WhatsApp social support group	Students were added to a WhatsApp group to: receive encouragement and support to exercise from the PEAK design team and provide/receive peer support from other students; share their experience of participating in exercise; co-ordinate times to meet up to exercise with peers; and ask the PEAK design team any questions about PEAK and/or exercise.	Digitally via WhatsApp	Week 1 - 12
“PEAK Packs” exercise group	Students had the option to take part in PEAK in a group called a ‘PEAK Pack’. These could be formed with existing friends, or by asking the program team to place a student in a group of demographically similar students. A separate WhatsApp groups was created for each PEAK Pack to help members coordinate with and support each other.	In person and digitally via WhatsApp	Week 1 - 12
Exercise and wellbeing trackers	Students were sent an “exercise tracker” each week to track the number, type and duration of exercise they completed.	Digitally via Qualtrics link embedded in text message	Week 1 - 12
	Students were sent a “wellbeing tracker” tri-weekly (i.e., once every three weeks) to track the impact exercise had on their mental wellbeing and cognition.	Digitally via Qualtrics link embedded in text message	Week 3, 6, and 9
Program outcome reports	A personalised “PEAK Outcome Report” was provided at the conclusion of the program. This report included the number of days the student had exercised per week; the total number of minutes spent exercising; and their mental, and cognitive health outcomes.	Digitally via email	Week 13

*Access video content from: https://youtube.com/playlist?list=PLHKkW53lhmzRRKdL8M5TyBoZiIAm-WrwY&si=akmm0QjyXtQbV5So. Adapted from Brown C, Richardson K, Halil-Pizzirani B, Hughes S, Atkins L, Perowne R, Pitt J, Yücel M, Segrave R. (2023) PEAK Mood, Mind and Marks: Development of an Intervention to Support University Students’ Mental and Cognitive Health through Physical Exercise. Manuscript in review for publication at BMC Public Health.

Neuroscience informed two key elements of the program: 1) incorporation of both moderate to vigorous intensity and resistance exercise options and 2) education about the brain, cognitive, and mental health mechanisms and impacts of these types of exercise. Specifically, each week, students were given access to a new menu of exercise options curated by a senior exercise physiologist (SH) that featured moderate to vigorous intensity aerobic and resistance exercises. Aerobic exercise options were purposefully selected with the physiological goal to elevate students’ heart rate. Resistance training options were purposefully selected with the physiological goal to systematically fatigue targeted muscle groups through applying force or weight. During the “Kick Off session” PEAK program onboarding and throughout the educational and motivational videos, students’ were educated about the positive brain effects of exercise and neuroscientific evidence that demonstrates exercise can optimise cognition and buffer stress to support academic performance. Students were also informed about evidence for two key neurobiological mechanisms (i.e., augmentation of BDNF and IGF-1 release) through which moderate to vigorous intensity exercise and resistance exercise can differentially support mental and cognitive health by changing the structure and function of the brain.

### 2.4 Behavioural and Health Outcome Measurement Procedures

Behavioural and health outcomes were tracked via self-report questionnaires sent by text message using the Qualtrics platform (see **Table 2** for data collection schedule). If students did not complete a survey within 24-hours, a single reminder text was sent.

**Table 2:** Behavioural and health outcome data collection tools and schedule.

Outcome	Scale/measurement tool	Collection schedule			
		Baseline	Weekly	Tri-weekly	End-point
Exercise					
Exercise frequency	Exercise tracker		X		
Exercise duration (minutes)	Exercise tracker		X		
Moderate-vigorous exercise	International Physical Activity Questionnaire	X			X
Sedentary behaviour	International Physical Activity Questionnaire	X			X
Mental health					
General mental wellbeing	Short Warwick-Edinburgh Mental Wellbeing Scale	X		X	X
Stress and coping	Perceived Stress Scale 4	X		X	X
Loneliness	Campaign to End Loneliness Tool	X		X	X
Psychological distress	Kessler Psychological Distress Scale	X			X
Cognitive health					
Concentration	VAS: Vague – Laser-like focus	X		X	X
Memory	VAS: Very forgetful – Rock-solid memory	X		X	X
Productivity	VAS: Unproductive – Highly productive	X		X	X
Belonging to the university					
Sense of belonging to the university	VAS: Very weak – Very strong	X		X	X

Exercise frequency and duration were measured each week for the duration of the program via a one-minute “exercise tracker” survey. All other behavioural and health outcomes were assessed at baseline (i.e., “week 0”) and at end-point (i.e., “week 12”) via a ten-minute assessment battery. Key mental and cognitive health outcomes and sense of belonging to the university were assessed once every three weeks via a four-minute “wellbeing tracker” assessment battery outlined below.

### 2.5 Participant characteristics

Information regarding students’ age, gender, domestic vs international student enrolment status, year of undergraduate degree, residential status (i.e., “off-campus” or “on-campus”), and current exercise engagement (i.e., whether they identified as a “non-exerciser”, “sometimes-exerciser” or “regular-exerciser”) were collected at baseline.

### 2.6 Behavioural and health outcomes

#### 2.6.1 Exercise frequency

The primary outcome variable was the proportion of students who engaged in three or more exercise sessions per week throughout the program, in keeping with the primary behavioural goal of the program. This was assessed by asking students to report the number of exercise sessions they engaged in each week via the “exercise tracker” survey.

#### 2.6.2 Exercise duration

Students were asked the number of minutes each exercise session they participated in each week via the “exercise tracker”. Students were told at the start of the program that an ‘exercise session’ was defined as ten-minutes or more of purposeful exercise.

#### 2.6.3 Moderate-vigorous exercise and sedentary behaviour

The International Physical Activity Questionnaire (IPAQ) (75) was used to assess (i) the number of hours spent engaging in moderate-vigorous exercise in the past week, and (ii) the number of hours spent sedentary on a typical weekday during the past week.

#### 2.6.4 Mental and cognitive health

Wellbeing was measured with the Short Warwick-Edinburgh Mental Wellbeing Scale (SWEMWBS) (76): The SWEMWBS is a seven-item measure of psychological wellbeing over the previous two weeks. It contains five response categories on a Likert-scale that are summed to provide a raw score that is transformed into metric scores ranging from 7–35.

Stress and coping were measured with the Perceived Stress Scale 4 (PSS-4) (77): The PSS-4 is four-item measure of the degree to which situations in one’s life are appraised as stressful and how well one feels they are coping with them over the past week. The scale has five response options on a Likert-scale which are summed to provide a score ranging from 0–16.

Loneliness was measured with the Campaign to End Loneliness Measurement Tool (CELMT) (78): The CELMT is a three-item measurement tool for assessing people’s experience of loneliness. Students were asked to answer each item using a five-point Likert scale. Scores are summed to give an overall loneliness score that ranges from 0 – 12.

Psychological distress was measured with the Kessler Psychological Distress Scale (K10) (79). The K-10 is a 10-item questionnaire intended to yield a global measure of distress based on questions about anxiety and depressive symptoms that a respondent has experienced in the most recent month. Students were asked to rate items on a five-point Likert scale. Response items are summed to provide a score ranging from 10 – 50.

Concentration, memory, and productivity was measured with Visual Analogue Scales (VAS): Students were asked to rate their concentration (from Vague to Laser-like focus), memory (from Very forgetful to Rock-solid memory), and productivity (from Unproductive to Highly productive) over the past week from 0–100 on a 100-point VAS.

#### 2.6.5 Belonging to the university

Sense of belonging to the university was measured with a Visual Analogue Scale (VAS): Students were asked to rate the strength of their sense of belonging to the university (from Very week to Very strong) over the past week from 0–100 on a 100-point VAS.

### 2.7 Implementation outcomes

Implementation outcomes included student engagement in PEAK, factors influencing engagement, the acceptability and appropriateness of PEAK, and mechanisms of behaviour change. The selection of these outcomes was based on common leading indicators of implementation success (80, 81).

#### 2.7.1 Participant engagement

Students’ engagement in each component of PEAK was measured via attendance records and digital analytics.

#### 2.7.2 Factors influencing engagement, intervention acceptability and appropriateness, and mechanisms of behaviour change

Semi-structured interviews were conducted with a subset of 15 students at the conclusion of the program to understand barriers to their engagement in PEAK, the acceptability and appropriateness of the program, and the mechanisms of behaviour change. Recruitment was stratified to obtain perspectives from students with low (n = 5) vs. moderate (n = 7) vs. high engagement in the program (n = 3), international (n = 8) vs. domestic (n = 7) students, and women (n = 8) vs. men (n = 7). The semi-structured interview schedule was guided by the COM-B model (see **Supplementary File 2** for interview guide). Questions were designed to investigate:

1. Factors influencing engagement (i.e., what helped and/or hindered students’ engagement in the program components)?
2. Intervention acceptability (i.e., how agreeable, palatable, or satisfactory were the program components) and appropriateness (i.e., how relevant, fitting, and compatible were the program components)?
3. Mechanisms of behaviour change (i.e., how did the program influence students’ capability, opportunity, and motivation to exercise)?

Interviews were conducted online via the Zoom platform (82), individually facilitated by authors (CB, RS or KR) and took approximately 40 - 60 minutes. Interviews were audio recorded and transcribed verbatim by a professional transcription service.

### 2.8 Data analysis

#### 2.8.1 Behavioural and health outcomes

Quantitative data was analysed using Statistical Package for Social Sciences (SPSS) version 29. Only participants who completed both the baseline and end-point assessments (n = 45) were included in the quantitative analyses. Descriptive statistics were used to characterise all behavioural and health outcomes and observe patterns of change in these variables across time. Paired-samples t-tests were used to investigate mean differences in moderate-vigorous exercise, sedentary time, mental and cognitive health outcomes, and sense of belonging to the university between baseline and end-point.

As students differed in how regularly they were exercising before beginning PEAK, an exploratory analysis was conducted to examine whether baseline exercise levels impacted engagement in moderate-vigorous exercise and sedentary behaviour from baseline to end-point. Students were grouped according to whether they identified as a “non-exerciser” (n = 11), “sometimes-exerciser” (n = 27) or “regular-exerciser” (n = 7) at baseline. Data were non-normally distributed among these sub-groups. As such, a series of Wilcoxon Signed-Rank tests were used to investigate potential differences in median moderate-vigorous exercise and sedentary behaviour between baseline and end-point among these groups. Alpha was set at.05, two-tailed for all analyses.

#### 2.8.2 Implementation outcomes

##### 2.8.2.1 Participant engagement

Descriptive statistics were used to characterise students’ engagement in each of the components of PEAK.

##### 2.8.2.2 Factors influencing engagement, intervention acceptability and appropriateness, and mechanisms of behaviour change

Transcripts were analysed using template analysis (83)—a thematic analytic method (84)—which permits a combination of both *a priori* themes relevant to the research question (e.g., whether PEAK impacted students’ capability, opportunity and motivation to exercise) alongside inductive coding to develop additional emergent themes. *A priori* themes were guided by the six categories of the COM-B model. While template analysis was conducted with a psychological lens, the eclectic research expertise of the authors provided an opportunity for input from a wider range of perspectives and disciplines (e.g., behavioural science, neuropsychology, clinical psychology, implementation science and evaluative synthesis).

Preliminary coding was conducted independently by two authors (CB, KR) using *a priori* themes to identify which COM-B categories were relevant to the design and/or implementation of PEAK. Codes were clustered into meaningful groups in which hierarchical and lateral relationships between themes were defined. The authors met to discuss coding of the two transcripts (designed to increase calibration between authors) and establish an initial template for coding the remaining transcripts. The initial template was further refined through systematic coding of the remaining transcripts conducted by two authors (CB and KR or BS or SH). The final template was re-applied to the full dataset by one author (CB).

## 3 Results

### 3.1 Participant characteristics

One hundred and fifteen undergraduate students signed up to the PEAK program pilot study. Of these, 74 (*M* age = 20.32, *SD* = 1.38 years; 46 women, 25 men, 4 non-binary gender) completed the online baseline assessment battery and their participant characteristics are detailed in **Table 3**. Students varied regarding their enrolment status (41 domestic, 33 international) and exercise history at baseline (21 “non-exercisers”, 38 “sometimes-exercisers”, 15 “regular-exercisers”).

**Table 3:** Participant characteristics.

Demographic	Frequency (%)
Gender	
Women	46(62)
Men	25(34)
Non-binary	4(5)
Enrolment status	
Domestic	41(55)
International	33(45)
Undergraduate year level	
First	14(19)
Second	21(28)
Third	30(41)
Fourth	5(7)
Fifth	4(5)
Residential status	
Off-campus	49(66)
On-campus	25(34)
Exercise history at baseline	
Non-exerciser	21(28)
Sometimes exerciser	38(51)
Regular exerciser	15(20)

### 3.2 Behavioural and health outcomes

#### 3.2.1 Exercise frequency

**Figure 1:**
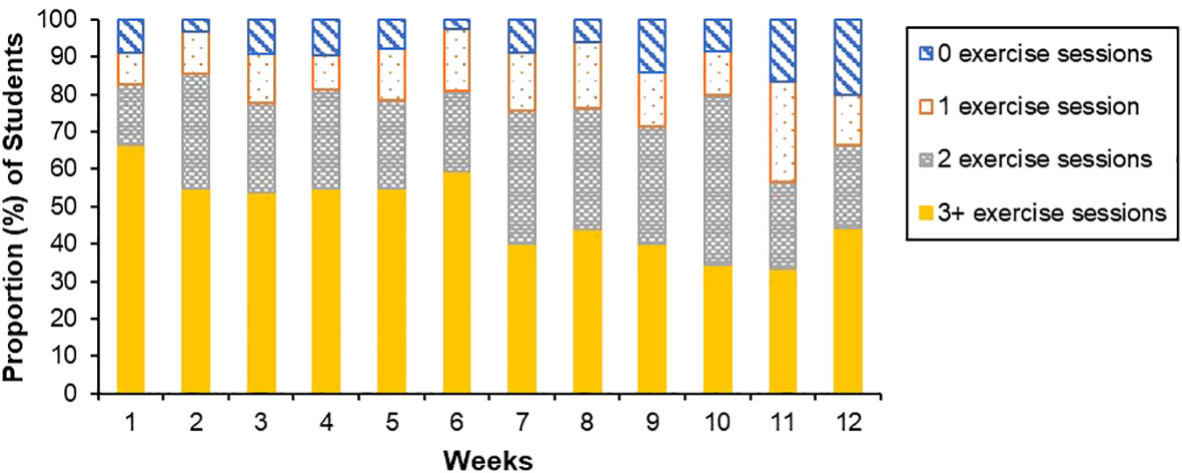
Average weekly number of exercise sessions. Week 12 = end-point.

Across the program, the average proportion of students who met the program target of exercising three or more times per week was 48.38% (*SD* = 10.49). When viewed week by week, the proportion of students engaging in three or more sessions per week decreased from week one (66.67%) to week 12 (44.44%) and fluctuated throughout the program (**Figure 1**). Of note, these figures are a conservative estimate as low engagement in the exercise tracker limited the amount of quantitative data available to evaluate exercise frequency (see **Table 4**). Qualitative findings revealed that students were often engaging in exercise sessions they did not record via the exercise tracker, due to the burdensome nature of the data collection method (see section 3.3.4.7).

**Table 4:** Student engagement in PEAK.

Engagement outcome	No. of participants (% of participants)
“Kick Off” program onboarding	
Total attendance	73(63)
Moodle delivery content	
Week 1	71(62)
Week 2	53(46)
Week 3	39(34)
Week 4	34(30)
Week 5	34(30)
Week 6	29(25)
Week 7	26(23)
Week 8	19(17)
Week 9	18(16)
Week 10	16(14)
Week 11	11(10)
Week 12	10(9)
Weekly average	30(26)
Total	108(94)
Monash Sport Passes	
Weekly average	14(12)
Total	45(39)
“PEAK Packs”	
Opted for “BYO PEAK Pack”	25(22)
Opted for “Fresh PEAK Pack”	14(12)
Total	39(34)
WhatsApp	
Read message	95(83)
Posted	33(29)
Exercise tracker	
Week 1	69(60)
Week 2	62(54)
Week 3	54(47)
Week 4	53(46)
Week 5	51(44)
Week 6	42(37)
Week 7	45(39)
Week 8	34(30)
Week 9	35(30)
Week 10	35(30)
Week 11	30(26)
Week 12	45(39)
Weekly average	46(40)
Total	68(59)
Wellbeing tracker	
Week 3	54(47)
Week 6	42(37)
Week 9	35(30)
Tri-weekly average	43(38)
Total	53(46)
“PEAK Points” and rewards	
Exchanged “PEAK Points” for coffee	23(20)
Exchanged “PEAK Points” for personalised small group exercise session	20(17)
Total	36(31)

The number of times each video was viewed varied across video topics, ranging from 58 total views for the video in week one to 10 total views for the video in week 12, with a higher number of views for videos presented earlier in the program (**Table 5**).

**Table 5:** Total number of educational and motivational video views.

Week	Video title	Total no. of views
Baseline	Welcome to PEAK	68
1	Expert tips for CREATING an exercise routine	58
2	Too many assignments to exercise? Exercising your way to PEAK marks	34
3	Overcoming self-consciousness	26
4	But, what if I don’t have time to exercise?!	26
5	Too stressed to exercise? Exercising your way to PEAK mood	22
6	Strong Lungs, Strong Brain: the beauty of breathlessness	21
7	Strong Body, Strong Brain: the beauty of strength training	21
8	Connecting with your WHY	19
9	Expert tips for SUSTAINING an exercise routine	16
10	Consistency trumps perfection, habits are the gold	9
11	But, what if I don’t feel like exercising?!	10
12	SWOTVAC is coming….Keep moving!	10

#### 3.2.2 Exercise duration

**Figure 2:**
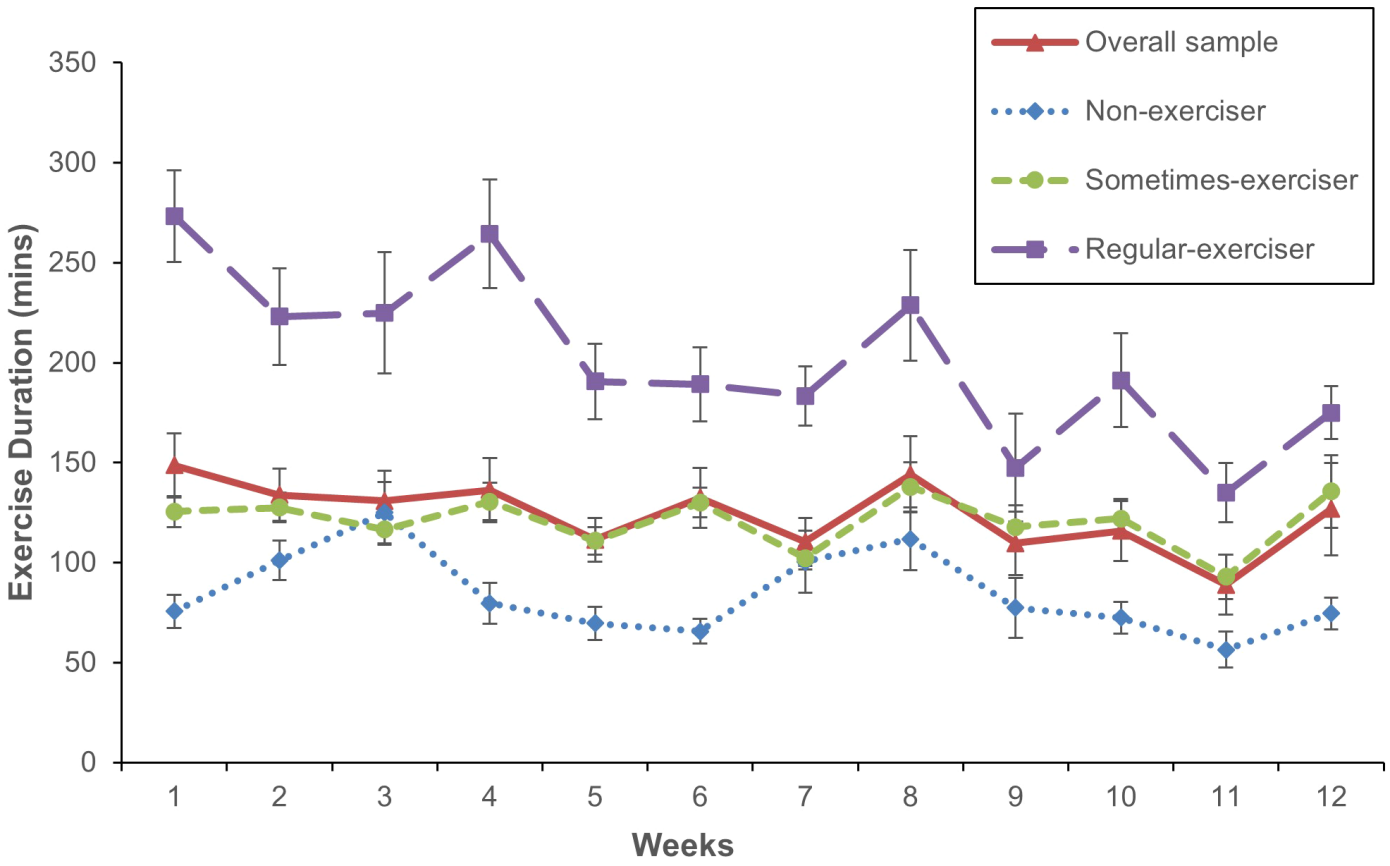
Mean weekly exercise duration throughout PEAK. Week 12 = end-point. Error bars represent standard error of the mean.

Students spent an average of 124.10 (*SD* = 17.16) minutes per week exercising throughout PEAK, with the cumulative duration decreasing gradually throughout the program from a mean of 148.61 minutes (*SD* =133.54) in week one to 126.78 minutes (*SD* = 153) in week 12 (**Figure 2**).

#### 3.2.3 Moderate-vigorous exercise and sedentary behaviour

**Figure 3:**
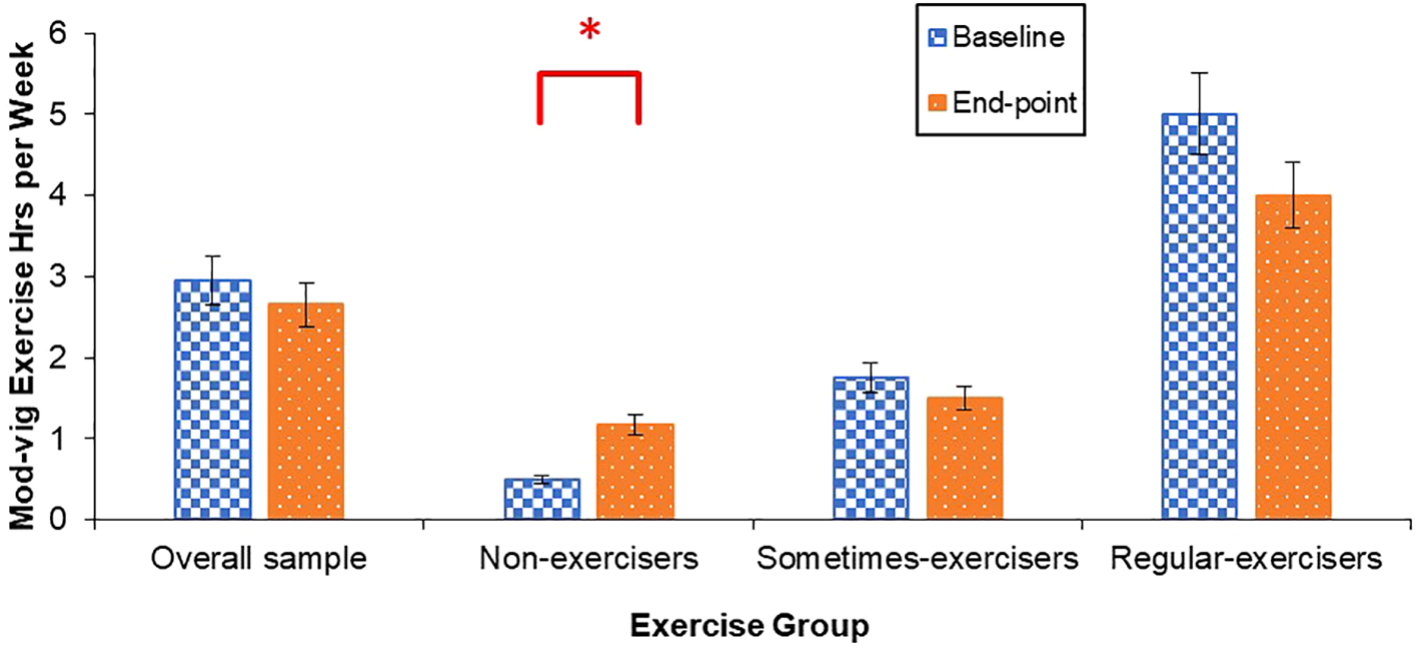
Mean hours spent engaging in moderate-vigorous exercise per week at baseline and end-point. Error bars for the overall sample represent standard error of the mean, and error bars for non-exercisers, sometimes-exercisers, and regular-exercisers represent interquartile range. *p <.05.

Students who identified as “non-exercisers” at the beginning of the program reported an increase in the number of hours spent engaging in moderate-vigorous exercise from baseline to end-point (*Z* = 2.30, *p* = .022, *r* =.49) (**Figure 3**). No change in moderate-vigorous exercise were observed for students who identified as “sometimes-exercisers” or “regular-exercisers”.

**Figure 4:**
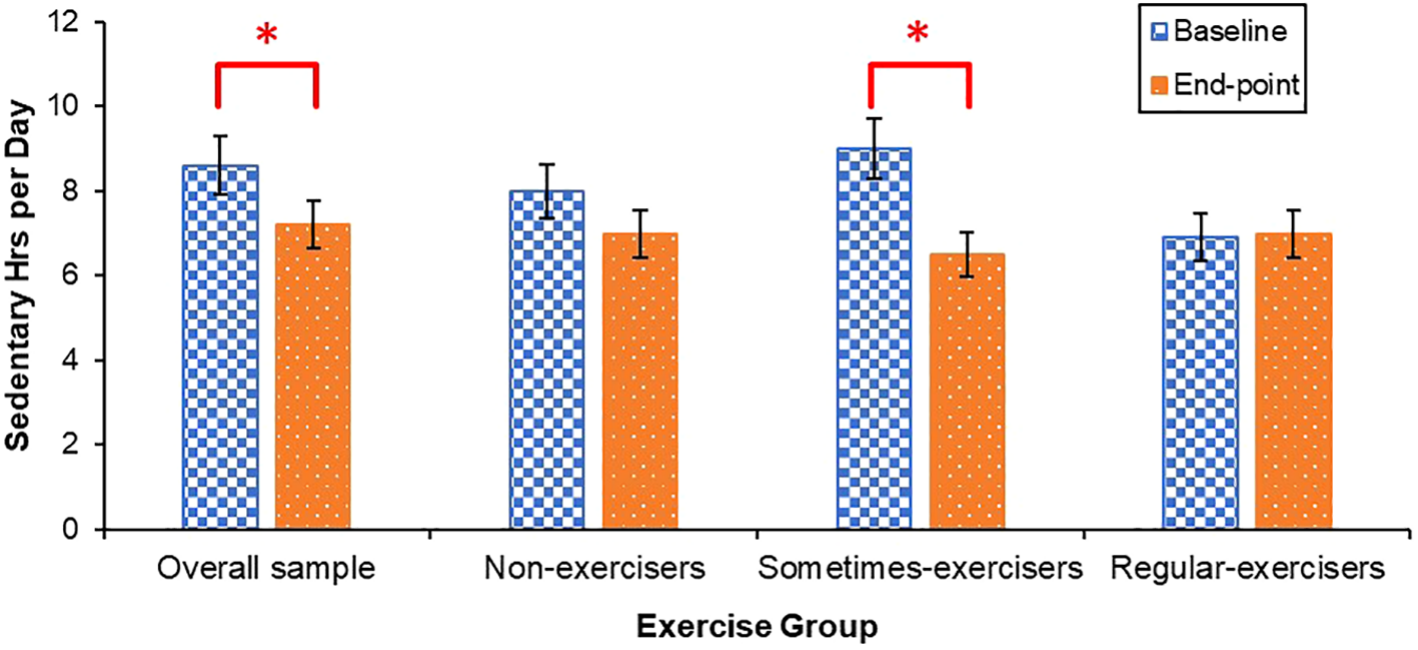
Mean daily sedentary behaviour at baseline and end-point. Error bars for the overall sample represent standard error of the mean, and error bars for non-exercisers, sometimes-exercisers, and regular-exercisers represent interquartile range. *p <.05.

Across all students, daily sedentary behaviour decreased from baseline to end-point, [*t* (44) = 2.38, *p* = .022, *Cohen’s d* = 3.94, 95% CI (.05,.65)] (**Figure 4**). Students who identified as a “sometimes-exerciser” at the beginning of the program also reported a decrease in daily sedentary behaviour (*Z* = 2.45, *p* = .014, *r* = .33). No change in daily sedentary behaviour were observed for students who identified as “non-exercisers” or “regular-exercisers”.

#### 3.2.4 Mental and cognitive health and sense of belonging to the university

**Figure 5:**
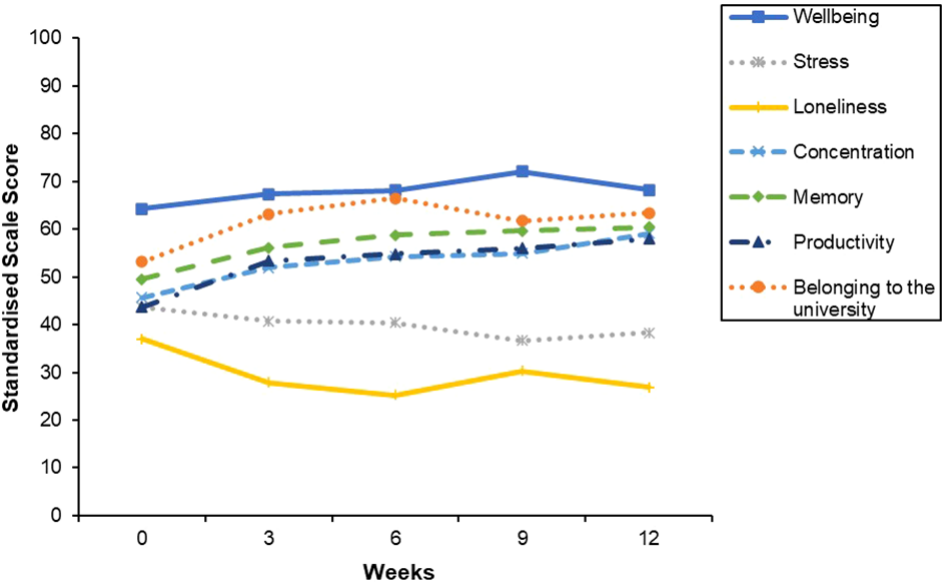
Mean mental wellbeing, stress, loneliness, concentration, Memory, Productivity and Belonging to the University throughout PEAK. Week 0 = baseline. Week 12 = end-point. Scale ranges were converted to 0–100 for all scales to enable standardised graph presentation.

There was a pattern of improvement in mental and cognitive health outcomes and students’ sense of belonging to the university throughout PEAK (**Figure 5**).

**Figure 6:**
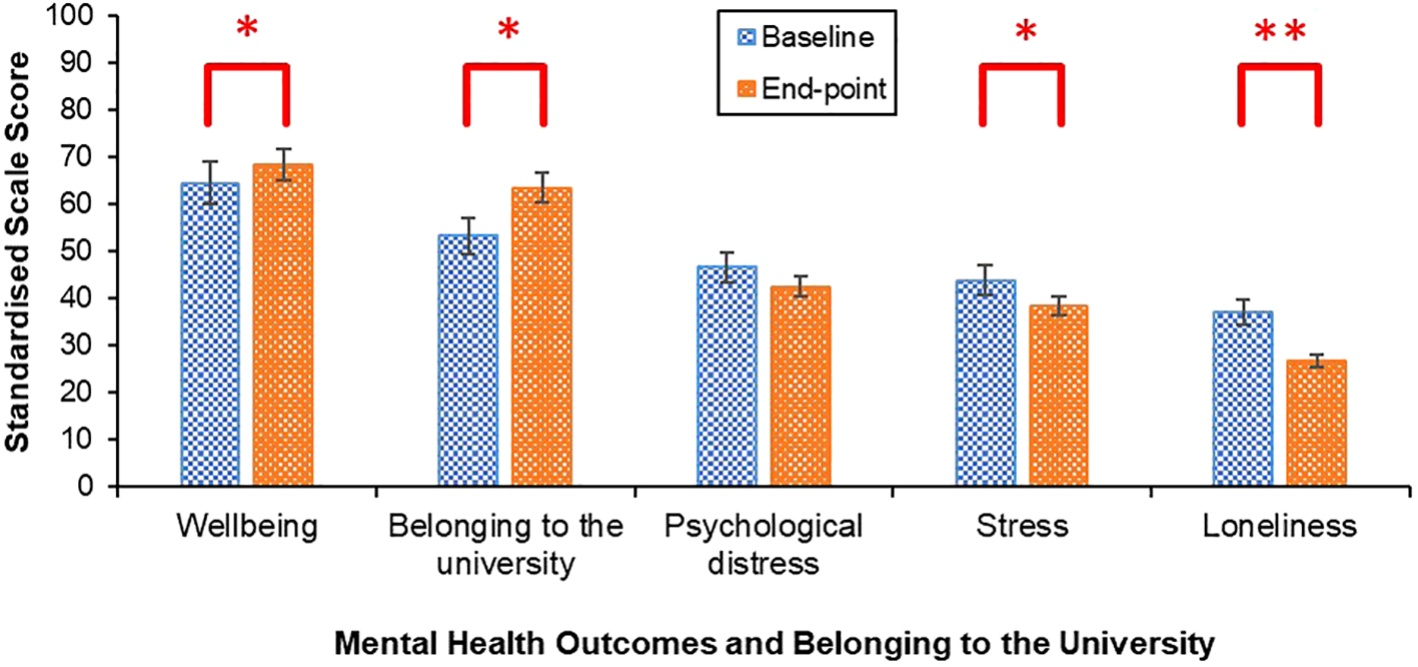
Mean mental wellbeing, belonging to the university, psychological distress, stress and loneliness scores at baseline and end-point. Error bars represent standard error of the mean. Scale sores were standardised by converting the scale range to 0–100 to enable standardised graph presentation. **p <.01, *p <.05.

Improvements in mental wellbeing [*t* (44) = 2.17, *p* = .035, *Cohen’s d* = 4.26, 95% CI (-.62, -.02)] and sense of belonging to the university were observed [*t*(44) = 2.59, *p* = .013, *Cohen’s d* = 26.54, 95% CI (-.69, -.08)], alongside decreases in stress [*t*(44) = 2.04, *p* =.048, *Cohen’s d* = 2.86, 95% CI (.003,.60)], and loneliness [*t*(44) = 3.24, *p* = .002, *Cohen’s d* = 2.53, 95% CI (.17,.79)] from baseline to end-point (**Figure 6**). Psychological distress did not significantly change [*t*(44) = 2.00, *p* = .051, *Cohen’s d* = 6.77, 95% CI (-.002,.60)].

**Figure 7:**
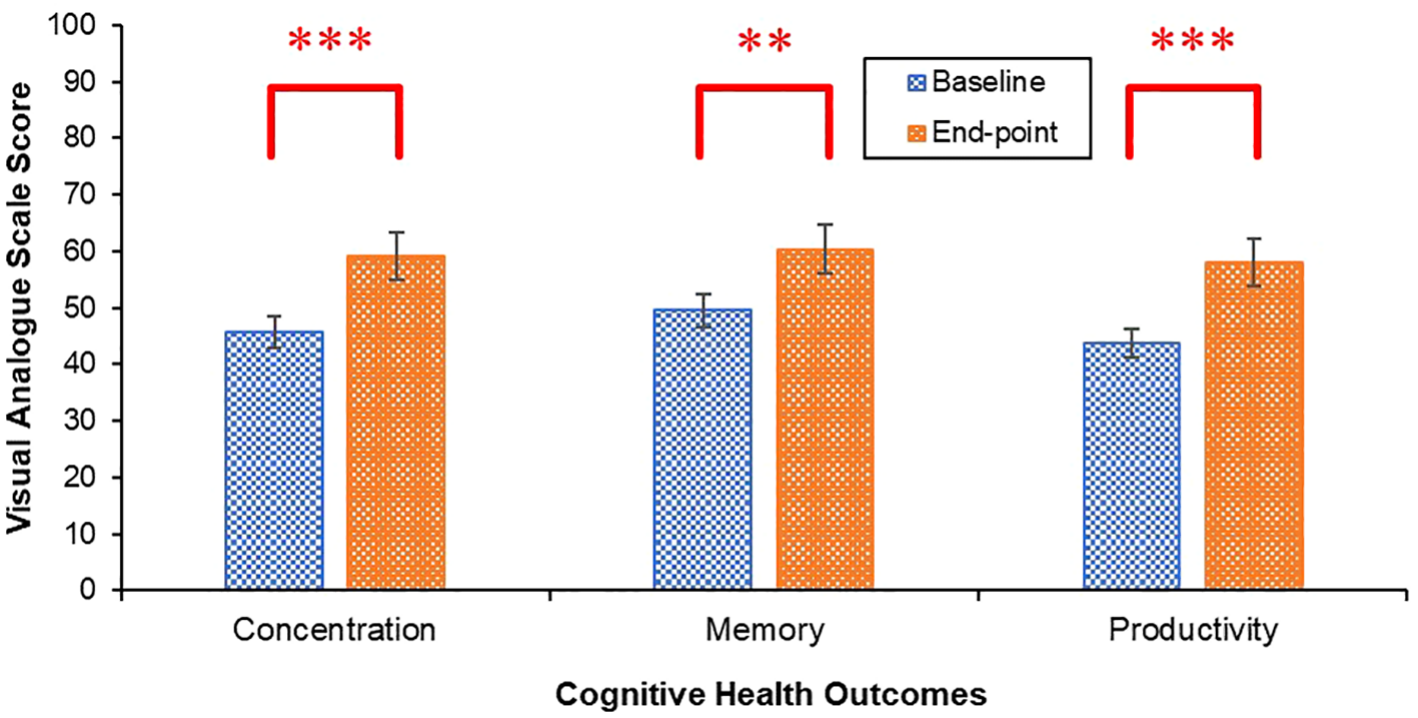
Mean concentration, memory and productivity scores at baseline and end-point. Error bars represent standard error of the mean. ***p <.001, **p <.01.

Improvements in perceived concentration [*t*(44) = 4.42, *p* <.001, *Cohen’s d* = 20.29, 95% CI (-.98, -.33)], memory [*t*(44) = 3.19, *p* = .003, *Cohen’s d* = 22.86, 95% CI (-.78, -.16)] and productivity [*t*(44)= 3.75, *p* <.001, *Cohen’s d* = 25.54, 95% CI (-.87, -.24)] were observed from baseline to end-point (**Figure 7**).

### 3.3 Implementation outcomes

#### 3.3.1 Participant engagement

As anticipated, engagement in the eight core components of PEAK fluctuated throughout the 12-week program (**Table 4**). Of the 115 students who enrolled, 108 (94%) engaged in one or more components during week one and 73 (68%) remained engaged in one or more components during week 12. Engagement in the weekly components of the program (i.e., the Moodle content and exercise trackers) progressively declined throughout the program. The number of students who accessed the Moodle content dropped from 71 (62%) during week one to 10 (9%) during week 12. The number of students who completed the exercise tracker also dropped from 69 (60%) during week one to 45 (39%) during week 12.

#### 3.3.2 Factors influencing engagement, intervention acceptability and appropriateness, and mechanisms of behaviour change

Qualitative interviews revealed several first and second level subthemes relevant to the three implementation outcomes: “factors influencing engagement”, “intervention acceptability and appropriateness”, and “mechanisms of behaviour change” (see **Supplementary File** for a complete list of themes and exemplar quotes).

#### 3.3.3 Factors influencing engagement

Students described several factors that influenced their engagement in PEAK including fluctuations in university workload, living off-campus, the timing of program components, cultural considerations, and time management.

##### 3.3.3.1 University workload

Students reported manageable academic demands at the start of PEAK, which coincided with the start of the university semester. As academic pressures intensified, in the lead up to the examination period (i.e., from week 9), engaging with the program, participating in exercise, and responding to the exercise and wellbeing trackers was deprioritised and more challenging to maintain.

##### 3.3.3.2 Living off-campus

For some, living off-campus negatively influenced program engagement. Some off-campus students felt disconnected from the program due to the emphasis on on-campus exercise options and rewards and the long commute required for them to access these. While they were aware of the off-campus program exercise options, namely the digital exercise options, these could have been made more prominent and no off-campus program rewards were offered.

##### 3.3.3.3 Scheduling conflicts

Scheduling conflicts due to lectures or work commitments prevented some students from being able to attend the “Kick Off” PEAK program onboarding session and, as such, they did not receive the same depth of program induction or have the opportunity to meet other PEAK participants in-person before PEAK started. Scheduling conflicts was also a barrier for some students who were not able to use their PEAK points to attend a small group exercise session.

##### 3.3.3.4 Time-management

Most students with low engagement in PEAK described a persistent struggle with time management throughout the program. Despite being aware of the important role time-management plays in initiating and maintaining an exercise routine, they reported poor time management skills impeded their ability to engage in PEAK and exercise.

#### 3.3.4 Intervention acceptability and appropriateness

Regardless of the degree to which they engaged, all students felt positive about the PEAK program and wanted it to continue in subsequent semesters. Some were enthused that the program symbolised the university genuinely values student mental and cognitive wellbeing, others about it being an exercise-based opportunity, and many by the novel combination of both. All students had suggestions of how to improve to the program for future iterations.

##### 3.3.4.1 “Kick Off session” program onboarding

Students who attended the onboarding session described it as informative, engaging and clearly conveyed what was involved in participating in PEAK. Learning about the program helped to inform students’ expectations about how to embed exercise in their routine from the outset.

##### 3.3.4.2 Menu of exercise options

The menu of exercise options was generally well-received by the students who found the content relevant, acceptable, and accessible. Some students noted the free passes to access the university gym were particularly important for their program engagement and ability to meet their exercise goals. The exercise starter kit also enabled student to engage in workouts at home if attending the gym was not accessible or appealing. However, some students found some items did not meet their needs or they already owned them. Some students would also seek out their own exercise options if the program’s recommendations did not include a specific exercise of interest, or if they found the exercise options too burdensome to access. For instance, some students found it tedious to log into Moodle, select the PEAK program icon, and open the menu of exercise options each day to view the content.

##### 3.3.4.3 Educational and motivational videos

Perspectives on the videos were mixed. While video viewership across the program cohort was low-moderate (with Moodle-based delivery and the weekly-release format being the biggest barriers to this), some of the students who viewed them described the content as engaging and particularly enjoyed those that reflected authentic student life, used humour, and/or prompted reflection on the reasons why exercising was important to them. Other students, including those who were less engaged in PEAK, did not find the videos acceptable or appropriate. These students either found the videos difficult to access online (i.e., involving too many steps), felt the video style was not engaging or the content was not relevant because they already knew about the benefits of exercise.

##### 3.3.4.4 PEAK points reward system

The concept of a points-based reward system was highly acceptable; however, the nature of the rewards and the PEAK points delivery system were felt to be sub-optimal. Students wanted the coffee vouchers to be redeemable at a more popular café than the student union cafeteria. They also wanted to redeem small group exercise sessions earlier in the 12-week program than was made possible by the points allocation system. The process to redeem rewards involved numerous steps: logging exercise sessions to receive points, checking text messages to check point balance, responding to a text message to redeem points, then either receiving a coffee voucher or booking into a small group training session, which students found unnecessarily cumbersome. As such, a sizable subset of students accumulated points without redeeming them.

##### 3.3.4.5 WhatsApp social support group

The acceptability of the WhatsApp peer-support group varied. For many students, the group fostered a sense of community, and they appreciated the exchange of exercise information, stories, and photographs. However, while all students had access to the group, only a small proportion actively engaged in conversation. Feeling overwhelmed by the volume of communications within the group, shyness, pressure to post a great message, and/or not having met most other members in person contributed to students’ reluctance to post a message. Students agreed that more opportunities to meet each other in-person would have increased their engagement in WhatsApp, and the program more broadly. Interestingly, many students who did not post on the WhatsApp feed continued to read it and described value in doing so as it gave them a sense of accountability and solidarity – even in the absence of sharing their own exercise stories.

##### 3.3.4.6 PEAK Pack exercise groups

Experience of the PEAK Pack exercise groups were mixed as some groups stayed connected while others disbanded soon after forming. For the groups that remained intact, PEAK Pack’s provided social accountability to exercise, which increased participation in exercise and helped students overcome common barriers such as feeling tired, busy, or stressed. The success of these groups often hinged on the leadership of one or more members who organised meetups and maintained regular communication, effectively serving as self-appointed “champions”. Some students noted that their initial enthusiasm for their PEAK Pack waned as members became less responsive over time. A minority of the students who did not sign up to join a PEAK Pack subsequently felt less integrated into the program when Packs were discussed on WhatsApp.

##### 3.3.4.7 Exercise and wellbeing trackers

Engagement in the exercise and wellbeing trackers was suboptimal (see **Table 4**) and all students described engaging in exercise sessions that they did not report via the tracker. Barriers to completing the trackers were doing something else when the text request was received and subsequently forgetting, perceiving the exercise tracker was burdensome, tedious and/or a low priority. This reduced the amount of quantitative data available to evaluate the weekly and tri-weekly behavioural outcomes, including the primary exercise outcome, and means that these behavioural outcomes of the program are likely underestimated. There was also confusion about what duration or type of exercise should be reported in the tracker. For example, students were not sure whether to report multiple short bouts of exercise, dispersed throughout the day, or leisurely walks, which often led to the omission of this exercise from the tracker. Some students also found the exercise tracker demotivating if it highlighted their inactivity, leading to feelings of guilt and inadequacy. Despite variable engagement, many students found receiving a weekly text to track their exercise aided accountability and was a helpful prompt to exercise.

The brevity of the exercise tracker questions were appreciated, and many wanted to see their exercise progress. Conversely, the wellbeing trackers were felt to be too long by many. A minority of students also expressed uncertainty about the interpretation of some items in the wellbeing trackers (e.g., frequency of depressive feelings where students of the threshold that would qualify as depressed), demonstrating the importance of defining such terms.

##### 3.3.4.8 Program outcome reports

PEAK program outcome reports were well received and the simple visual representations of progress, opportunity to see improvements over time, and the sense of accomplishment these conferred were popular with most students. Knowing that they would receive a report at the conclusion of PEAK motivated some students to complete the exercise and wellbeing trackers and to maintain their engagement in exercise. However, a significant portion of students were unaware or had forgotten reports would be issue, and this negatively impacted their engagement with the exercise and wellbeing trackers.

##### 3.3.4.9 Moodle digital delivery platform

The use of Moodle as a digital delivery platform was controversial. Some students found it convenient to have PEAK integrated with their primary university learning system. Others found the platform too formal, disliked PEAK being associated with their academic work, avoided Moodle (and therefore PEAK content) when study stress was high, and/or found access burdensome due to log in requirements and incompatibility with mobile phones.

##### 3.3.4.10 Cultural considerations

Some international students suggested ways the cultural inclusivity of PEAK could be enhanced, primarily by providing exercise content in multiple languages and via digital platforms commonly used in their cultural groups. For example, an international student from China described using Keep, an exercise app developed in China, instead of the digital exercise options recommended in PEAK because she felt more comfortable using an app that could guide her through exercises in Chinese.

#### 3.3.5 Mechanisms of behaviour change

##### 3.3.5.1 Psychological Capability

As a result of participation, increases in knowledge were described across the following domains: the long-term benefits of consistently engaging in exercise, how exercise can improve mental, cognitive and brain health, and the variety of types of exercises that can be incorporated into an exercise regime. Students’ especially valued learning from student peers, especially those who were more experienced exercisers, and the PEAK program team, especially the exercise physiologist.

##### 3.3.5.2 Physical Opportunity

Easy access to a variety of exercise options enabled some students to engage in a wider variety of exercises. Students appreciated the flexibility to engage with the exercise content through on-campus and/or digital formats. The fact all exercise options were free of cost was a major enabler, and the provision of 12 free gym passes was especially useful.

Integrating PEAK into the Moodle platform, receiving a reminder text message to complete the exercise tracker, and seeing items from the exercise starter kit in their physical environment served as a helpful prompt and cue to exercise for a minority of students.

##### 3.3.5.3 Social Opportunity

The social components of PEAK were popular and enhanced accountability and enjoyment of exercise, and for many students, how hard they pushed themselves during sessions. Students valued the opportunity to form a community of student peers, and to form and strengthen friendships, especially for the purpose of exercising together.

The inclusive nature of the PEAK community, suited to those with varying levels of exercise experience and fitness, provided a welcoming environment to exercise. This was particularly appreciated by students who were new to exercise, felt too intimidated to exercise, or lacked a pre-existing group of friends to exercise with.

##### 3.3.5.4 Automatic Motivation

Many students described how they transitioned from viewing exercise as an obligation to a source of enjoyment as a result of taking part in PEAK. They also described the program helped them to establish a consistent exercise routine, which in turn contributed to a sense of accomplishment and wellbeing.

Engagement in exercise was reinforced by experiencing the beneficial impacts on their mental, cognitive, and physical health. These included improvements in: improvements in mental clarity, emotion regulation, mood, resilience, energy, ability to cope with stress, particularly in academically demanding periods, concentration, productivity, and physical fitness.

For some, the primary motivation to engage in exercise shifted from an extrinsic purpose, such as to lose weight, to an intrinsic purpose, such as enjoying how exercise made them feel.

##### 3.3.5.5 Reflective Motivation

Motivation to exercise was strengthened by the credibility of the program content and team, and consistent messaging about how exercise can support students’ to study well, alongside the mental, cognitive, and brain health benefits of exercise.

The PEAK Points reward system incentivised some students to engage in exercise, particularly during the initial stages of the program. Among these students, the appeal of earning points was initially a stronger motivator than the mental and cognitive health benefits of exercise.

The goal of exercising three times a week helped some students develop a regular exercise routine. The broad program goals to support students’ “mood, mind and marks” also resonated with students’ aspirations. Students felt the program content was appropriately tailored to student life and common student barriers and facilitators to exercise.

## 4 Discussion

The PEAK Mood, Mind, and Marks program was developed via consultation with university students, staff and senior leadership, application of the BCW (63) and informed by exercise neuroscience to support university students’ engagement in regular exercise for the betterment of their mental and cognitive health. To our knowledge, this program is the first of its kind to embed neuroscience-informed program content and messaging to target students’ barriers and facilitators to exercise, including their goals to excel academically. The current study was a pilot evaluation of PEAKs first iteration. Students’ engagement in exercise did not shift substantially from the beginning to the end of the program. All aspects of students’ mental and cognitive health improved, except psychological distress which remained stable. Overall, students were enthusiastic about an exercise-based program to support their mental and cognitive wellbeing and identified numerous ways to optimise the design and delivery of PEAK to better meet their needs and enhance their engagement.

On average, the exercise tracker data indicated that less than half of students met the program target of exercising three or more times per week, and this proportion decreased from the start to the end of the program. However, engagement with the weekly exercise tracker was low and likely to have led to an underestimation of students’ exercise engagement. Students perceived the exercise tracker as tedious and/or often forgot to complete the tracker. The explanation provided to students about what type and level of exercise could be logged was also unclear. Qualitative findings revealed students were often engaging in exercise that was not captured by the exercise tracker. In light of these limitations, it is difficult to discern with certainty the program’s impact on the frequency of students’ weekly exercise activity. These findings underscore the need for more accurate and less burdensome data collection procedures to be implemented moving forward. Given the proliferation of smartphones and personal wearable devices, an alternative solution for future evaluations of PEAK is the development of a mobile application that can be linked to a wearable device and incorporates passive activity sensing technology, providing an objective measure of exercise. Adopting such a measure would likely improve the accuracy of the exercise data collected and enhance participant engagement by removing the burden of self-report measures (85).

Although the weekly exercise data was unreliable, qualitative finding revealed students did experience ongoing challenges with prioritising exercise, particularly as the semester progressed and academic workload increased. Therefore, while the program may have helped some students initiate an exercise routine, maintaining this routine amidst competing academic priorities remained difficult. Further, students’ engagement in PEAK decreased throughout the program, which likely attenuated the program’s impact on maintaining students’ engagement in exercise at a whole group level. Interestingly students sedentary time decreased and students who identified as “non-exercisers” before PEAK increased their engagement in moderate to vigorous exercise. It is possible reducing sedentary behaviour was a more achievable behavioural goal for students, especially during the exam preparation period. For example, reducing sedentary behaviour can require less effort, time and resources than engaging in exercise (86–88). The increased engagement in moderate to vigorous exercise observed in non-exercises may reflect that these students were not engaged in exercise before participating in PEAK, and, therefore, have a greater scope for improvement compared to students who were already exercising (89).

While the impact of PEAK on students’ weekly exercise engagement was inconclusive, mental and cognitive health outcomes improved from the beginning to the end of PEAK, except for psychological distress, which remained stable. Students sense of belonging to the university also improved. Improvements in mental and cognitive health outcomes may have occurred due to an increase in exercise that was not captured by the exercise data collection method. While this would be in keeping with evidence demonstrating regular exercise can positively impact these outcomes (90–94), this conclusion is precluded by the absence of an observed increase in exercise activity and challenges with data collection. Alternatively, improvements may have occurred independent of any change in exercise, raising questions about the mechanisms driving these changes. Feeling part of a student peer collective outside of a classroom context may have strengthened many aspects of students’ social connectedness, which is a well-established determinant of mental health (95) and supports cognitive functioning (96). Indeed, the largest improvements were observed in loneliness and sense of belonging to the university relative to any other mental health outcomes, suggesting PEAK had a particularly positive impact on social wellbeing. Furthermore, the majority of the students interviewed reported PEAK had pervasive positive impacts on their social connectedness. Specifically, students’ felt PEAK help them to feel part of a community and strengthened their social support. These findings demonstrate PEAK had a particularly positive impact on students’ social wellbeing, which may have contributed to improvements in their mental and cognitive health.

All students who took part in the qualitative evaluation wanted PEAK to continue being offered at the university, even those with relatively low engagement in exercise and/or the program components. Students’ felt such a program could deliver substantial mental, cognitive, and physical health benefits to the student body, address a need to help students incorporate exercise into their daily lives, and demonstrated the university was genuinely invested in students’ mental and cognitive wellbeing. These perspectives are consistent with findings from the PEAK co-design process (63), in which students indicated a strong appetite for proactive exercise-based approaches to support their mental and cognitive health. Prior research has similarly reported that the acceptability of physical activity as a mental health intervention strategy within a university context is high (97), demonstrating that it is valuable to continue to develop these kinds of programs. While the appetite for programs like PEAK was high and students felt positive about the types of interventions delivered in the program (i.e., information about the neuroscientific mental and cognitive health benefits of exercise, identifying with the program goal to support their “mood, mind, and marks”, social support, flexible exercise options, credible content, reminders and rewards to exercise, self-monitoring and feedback on exercise engagement and its impacts on mental and cognitive health), they identified numerous opportunities to refine PEAKs content, delivery and design.

Students expressed a desire for more opportunities to connect with each other in-person and to improve the program’s cultural inclusivity. International students spoke to the need for digital exercise options to be expanded to include phone applications developed in a variety of cultural contexts. They also suggested to provide online exercise videos in multiple languages. These additions to the program content would enhance the cultural appropriateness of PEAK to better cater to a culturally diverse student population and align with the broader goals of equity and inclusion in public health interventions. Moreover, utilising a multichannel approach comprised of freely available digital channels (e.g., WhatsApp, Moodle, text message) to deliver the program components, while cost effective, did not optimally engage students. This approach was viewed as burdensome and overly complex, which reduced engagement. Students felt overwhelmed by the WhatsApp content due to the high volume of communications within the WhatsApp group and the different steps involved in accessing the program content via Moodle, responding to text messages to exchange their “PEAK Points” for rewards and/or completing the survey links sent to them via text message were convoluted. Developing PEAK into a single, unified digital delivery tool, such as a mobile application or website, could address this challenge. A single digital platform would streamline program delivery, making it more user friendly, and improve overall engagement and outcomes. Such consolidation could also reduce the staff resources required to support the program, thereby enhancing the scalability and implementation of PEAK within the university setting.

Finally, the multicomponent design could be enhanced by personalising the program content to students’ individual needs. As expected, students had varying opinions on whether they liked or disliked the same program component, influenced by their unique needs, priorities, preferences, and expectations. Therefore, students engaged with the program components that suited their needs most, and disengaged with the components that did not. A personalised program design could more effectively cater to the diverse needs of students, including international students. However, creating a personalised program required a level of complexity, resource, and financial input that could not feasibly be accommodated in the first iteration of PEAK. Given advances in technology such as artificial intelligence (AI) is developing rapidly, it is possible the pathway towards program personalisation may become more accessible and feasible. For instance, AI algorithms can be leveraged to analyse participants data, including demographics, fitness levels, and barriers and facilitators to exercise, to develop personalised programs that cater to people’s unique exercise needs, goals, and barriers to behaviour change (98, 99). Using AI in these ways to optimise program personalisation holds substantial potential to improve user acceptability, engagement, and overall impact on health outcomes (100). However, more research is needed to evaluate the effectiveness of AI-integrated programs in achieving these outcomes, both in a university setting and beyond.

### 4.1 Strengths and Limitations

The current study is the first to evaluate a BCW-informed health behaviour change intervention designed to support university students’ mental and cognitive health through exercise. The findings demonstrate clear benefits in conducting small scale pilot studies to optimise and refine behavioural interventions before expending significant resources on large scale clinical trials (69–72). The use of mixed-methods and a hybrid evaluation of the program’s preliminary behavioural and health impacts and implementation provides a comprehensive understanding of its potential effects and user experience. A key strength of the intervention was that it was designed with the intention of maximising its scalability by leveraging digital tools to deliver most of the intervention components.

The study data should be considered in light of several limitations. The evaluation methods were not able to assess the individual impacts of the eight program components and, as such, it is not possible to identify which components are essential to retain in PEAK to maximise program engagement and exercise outcomes. Moreover, standardised measures of students’ cognitive health and sense of belonging to the university were not used in the current evaluation, which may have affected corresponding findings. Additionally, the development and evaluation of PEAK focused on an Australian metropolitan tertiary education context. As such it is unclear the extent to which the findings from these studies generalise to universities in regional and remote settings, and tertiary education settings across geographically and culturally diverse countries. Lastly, while single-arm study designs are appropriate for early-stage pilot studies (101), the lack of a control or comparator group precludes conclusive determination of changes observed in behavioural and health outcomes to students’ engagement in PEAK. However, students’ strong appetite for programs of this nature coupled with the learnings about the program components provides a solid foundation upon which to optimise the program design and continue its dissemination and iteration.

### 4.2 Conclusion

The current pilot study demonstrated that an exercise-based approach to support students’ mental and cognitive health is acceptable. Students were especially receptive to the flexible exercise options, social components of the program, and the program rewards. Moreover, they valued the neuroscience-informed approach to motivational and educational content and that the program’s stated outcomes aligned with their academic goals. As is expected for the first pilot of PEAK, the program in its current form requires iteration to strengthen its impact, enhance student engagement, and improve its sustainability and scalability. A streamlined delivery format and personalised program content provide opportunities for optimising PEAK in these ways. Students suggested future iterations should include more opportunities to connect with student peers in-person and culturally inclusive content, demonstrating the potential for program optimisation. Overall, the current findings lay the groundwork for future research and refinement to optimise an impactful, scalable intervention to foster healthy exercise habits in university settings.

## Data availability statement

The raw data supporting the conclusions of this article will be made available by the authors, without undue reservation.

## Ethics statement

This study was approved by Monash University Human Research Ethics Committee (#32395). This study was conducted in accordance with the local legislation and institutional requirements. The participants provided their written informed consent to participate in this study.

## Author contributions

CB: Conceptualization, Data curation, Formal analysis, Funding acquisition, Investigation, Methodology, Project administration, Resources, Writing – original draft, Writing – review & editing. KR: Conceptualization, Data curation, Formal analysis, Funding acquisition, Investigation, Methodology, Project administration, Resources, Supervision, Writing – review & editing. B-HP: Conceptualization, Data curation, Formal analysis, Investigation, Methodology, Project administration, Resources, Supervision, Writing – review & editing. SH: Data curation, Investigation, Methodology, Resources, Writing – review & editing. LA: Methodology, Supervision, Writing – review & editing. JP: Investigation, Methodology, Project administration, Resources, Writing – review & editing. MY: Funding acquisition, Writing – review & editing. RS: Conceptualization, Data curation, Formal analysis, Funding acquisition, Investigation, Methodology, Project administration, Resources, Supervision, Writing – review & editing.

## Footnotes

1 Exercise is defined as “planned, structured, and repetitive bodily movement done to improve or maintain one or more components of physical fitness” (5).
