# Supplementary material for: PEAK Mood, Mind, and Marks: a pilot study of an intervention to support university students’ mental and cognitive health through physical exercise
Source: Front Psychiatry. 2024 Jun 10;15:1379396. doi: 10.3389/fpsyt.2024.1379396 (PMC11194434; doi:10.3389/fpsyt.2024.1379396)
Supplement: Supplementary file 1 [file DataSheet_1.docx]

**Supplementary Material**

| Table of contents | Page Number |
| --- | --- |
| Supplementary File 1. The TIDieR (Template for Intervention Description and Replication) Checklist | 2 |
| Supplementary File 2. PEAK Implementation Evaluation Interview  Schedule | 5 |
| Supplementary File 3. Implementation Evaluation Themes with Exemplar Quotes | 8 |

**Supplementary File 1**

**Table 1**

**
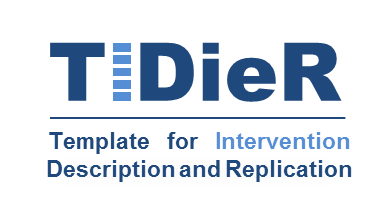
The TIDieR (Template for Intervention Description and Replication) Checklist*:**

Information to include when describing an intervention and the location of the information

| **Item number** | **Item** | **Where located **** | |
| --- | --- | --- | --- |
|  |  | Primary paper  (page or appendix  number) | Other ^†^ (details) |
|  | **BRIEF NAME** |  |  |
| **1.** | Provide the name or a phrase that describes the intervention. | Section 1 | ______________ |
|  | **WHY** |  |  |
| **2.** | Describe any rationale, theory, or goal of the elements essential to the intervention. | Section 1 & 2.3, Table 1 | Brown et al., (in review) |
|  | **WHAT** |  |  |
| **3.** | Materials: Describe any physical or informational materials used in the intervention, including those provided to participants or used in intervention delivery or in training of intervention providers. Provide information on where the materials can be accessed (e.g. online appendix, URL). | Section 2.3, Table 1 | Brown et al., (in review) |
| **4.** | Procedures: Describe each of the procedures, activities, and/or processes used in the intervention, including any enabling or support activities. | Section 2.3, Table 1 | Brown et al., (in review) |
|  | **WHO PROVIDED** |  |  |
| **5.** | For each category of intervention provider (e.g. psychologist, nursing assistant), describe their expertise, background and any specific training given. | Section 2.3, Table 1 | Brown et al., (in review) |
|  | **HOW** |  |  |
| **6.** | Describe the modes of delivery (e.g. face-to-face or by some other mechanism, such as internet or telephone) of the intervention and whether it was provided individually or in a group. | Section 2.3, Table 1 | Brown et al., (in review) |
|  | **WHERE** |  |  |
| **7.** | Describe the type(s) of location(s) where the intervention occurred, including any necessary infrastructure or relevant features. | Section 2.3 | Brown et al., (in review) |
|  | **WHEN and HOW MUCH** |  |  |
| **8.** | Describe the number of times the intervention was delivered and over what period of time including the number of sessions, their schedule, and their duration, intensity or dose. | Section 2.1 & 2.3, Table 1 | Brown et al., (in review) |
|  | **TAILORING** |  |  |
| **9.** | If the intervention was planned to be personalised, titrated or adapted, then describe what, why, when, and how. | N/A | N/A |
|  | **MODIFICATIONS** |  |  |
| **10.^ǂ^** | If the intervention was modified during the course of the study, describe the changes (what, why, when, and how). | N/A | N/A |
|  | **HOW WELL** |  |  |
| **11.** | Planned: If intervention adherence or fidelity was assessed, describe how and by whom, and if any strategies were used to maintain or improve fidelity, describe them. | Section 2.7 | _____________ |
| **12.^ǂ^** | Actual: If intervention adherence or fidelity was assessed, describe the extent to which the intervention was delivered as planned. | Section 3.3, Table 4 & 5 | _____________ |

** **Authors** - use N/A if an item is not applicable for the intervention being described. **Reviewers** – use ‘?’ if information about the element is not reported/not sufficiently reported.

† If the information is not provided in the primary paper, give details of where this information is available. This may include locations such as a published protocol or other published papers (provide citation details) or a website (provide the URL).

ǂ If completing the TIDieR checklist for a protocol, these items are not relevant to the protocol and cannot be described until the study is complete.

* We strongly recommend using this checklist in conjunction with the TIDieR guide (see *BMJ* 2014;348:g1687) which contains an explanation and elaboration for each item.

* The focus of TIDieR is on reporting details of the intervention elements (and where relevant, comparison elements) of a study. Other elements and methodological features of studies are covered by other reporting statements and checklists and have not been duplicated as part of the TIDieR checklist. When a **randomised trial** is being reported, the TIDieR checklist should be used in conjunction with the CONSORT statement (see [www.consort-statement.org](http://www.consort-statement.org)) as an extension of **Item 5 of the CONSORT 2010 Statement.** When a **clinical trial** **protocol** is being reported, the TIDieR checklist should be used in conjunction with the SPIRIT statement as an extension of **Item 11 of the SPIRIT 2013 Statement** (see [www.spirit-statement.org](http://www.spirit-statement.org)). For alternate study designs, TIDieR can be used in conjunction with the appropriate checklist for that study design (see [www.equator-network.org](http://www.equator-network.org)).

**Supplementary File 2**

PEAK Implementation Evaluation Interview Schedule

**Factors Influencing Engagement and Intervention Acceptability and Appropriateness**

**“Kick Off” session PEAK program onboarding & exercise starter pack**

1. How did you feel about the PEAK Kick Off welcome session?

- Did you attend a PEAK Kick Off welcome session?
  - Why / why not?

Probe for:

- - 1. knowledge + skills;
    2. accessibility + social influences;
    3. attitude + beliefs; habits + emotions

2. How did you feel about the exercise starter pack?

- Did you use the exercise starter pack?
  - Why / why not?

Probe for:

- - 1. knowledge + skills;
    2. accessibility + social influences;
    3. attitude + beliefs; habits + emotions

**Moodle delivery platform + content**

3. How did you feel about Moodle being the main delivery platform for PEAK?

- Did you engage in the Moodle content?
  - Why / why not?

Probe for:

- - 1. knowledge + skills;
    2. accessibility + social influences;
    3. attitude + beliefs; habits + emotions

4. How did you feel about the weekly “Learn a Bit” educational videos?

- Did you watch the videos?
  - Why / why not?

Probe for:

- - 1. knowledge + skills;
    2. accessibility + social influences;
    3. attitude + beliefs; habits + emotions

5. How did you feel about the ‘‘Move Some More” exercise options?

- Did you use the weekly “Move Some more” exercises?
  - Why/ why not?
- Did you use any of the free on-campus gym passes?
  - Why / why not?

Probe for:

- - 1. knowledge + skills;
    2. accessibility + social influences;
    3. attitude + beliefs; habits + emotions

**WhatsApp group/PEAK Pack exercise groups**

6. How did you feel about the PEAK WhatsApp chat?

- Did you read the WhatsApp chat (i.e., look at the chat)?
- Did you post on the WhatsApp chat? (i.e., emoji react to posts or post content)?
  - Why / why not?

Probe for:

- - 1. knowledge + skills;
    2. accessibility + social influences;
    3. attitude + beliefs; habits + emotions

7. How did you feel about the PEAK Packs?

- Did you sign up for a PEAK Pack?

1. Why / why not?

- **[If relevant]** How did the PEAK Pack work out for you?

Probe for:

- - 1. knowledge + skills;
    2. accessibility + social influences;
    3. attitude + beliefs; habits + emotions

**Tracking: exercise tracker + wellbeing tracker**

8. How did you feel about the survey trackers (i.e. your exercise tracker survey and wellbeing tracker survey)?

- Did you do any of the trackers?
  - How many?
  - Why / why not?
- Did you do exercise that wasn’t captured in the tracker survey?
- Did receiving a text to track your exercise each week impact how active you were?
  - Why / why not?
- Did knowing you would receive a report of your tracking data at the end of PEAK influence you doing the trackers (or not)?
  - Why / why not?
- How did you feel about the outcome report?

Probe for:

- - 1. knowledge + skills;
    2. accessibility + social influences;
    3. attitude + beliefs; habits + emotions

**“PEAK Points” rewards (coffees + personalised exercise sessions with exercise physiologist)**

9. How did you feel about the PEAK Points?

- Did you cash in your points for coffee vouchers or personalised exercise sessions?
  - Why / why not?

Probe for:

- - 1. knowledge + skills;
    2. accessibility + social influences;
    3. attitude + beliefs; habits + emotions

**Mechanisms of Behaviour Change**

11. Do you think you think you got any benefits out of PEAK? (Reflective Motivation)

- What kind?

12. Did you learn anything from PEAK that you didn't know at the start? (Psychological Capability)

13. Did you feel physically able to do the exercise activities? (Physical Capability)

14. Did PEAK impact your motivation to exercise? (Reflective Motivation)

15. Did PEAK impact your enjoyment of exercise? (Automatic Motivation)

16. Did PEAK impact your desire to exercise with others? (Social Opportunity)

17. Did PEAK impact your ability to fit exercise into your daily routine? (Physical Opportunity)

## **Supplementary File 3**

## **Table 2**

## *Implementation Evaluation Themes with Exemplar Quotes*

| Theme | Sub-theme 1 | Sub-theme 2 | Exemplar quotes |
| --- | --- | --- | --- |
| Factors influencing engagement | | | |
| Fluctuations in university workload | | | “From week 1 to week 7, I felt like my course content and the way my lectures and every - my assessments were structured, week 1 to week 7 was actually not that hectic. Then after week 7, things just got very, very hectic. I had so many assignments, so many things to do, and then I would keep procrastinating on exercising. I'd just be like, I'll do it later, I'll do it later, and then never eventually got to it.” (P005, female, international student, low engagement)  “Then I think, occasionally later on with uni assignments – I guess it’s just a matter of maybe I didn’t necessarily make it a priority to fill the surveys in” (P099, male, domestic student, moderate engagement) |
| Living off-campus | | | “I live a little far from campus. it's like an hour travel, so it's kind of just like I felt a little not engaged with the whole program at times in terms of - because I remember them giving access to 12 gym sessions at the Monash gym, and I didn't use any of them because I live so far away.” (P005, female, international student, low engagement) |
| Scheduling conflicts | | | “I didn't go [to the Kick Off session] because I work nine til five most days and there was no times that worked.” (P072, female, domestic, low engagement)  “I didn’t attend many of those [personalised small group training] sessions... I think with the timing – so it was a bit hard to always attend some of those training sessions.” (P099, male, domestic student, moderate engagement) |
| Time management | | | “it’s quite honestly about our time management and we don’t do that quite well. Even if we know that we don’t manage our time quite well, we’re still not going to do it next time either.” (P113, male, international student, moderate engagement) |
| Intervention acceptability and appropriateness | | | |
| Students perceive it is important for the PEAK program to continue to be offered at the university | | | “Having a program like this I think shows that there is still a lot of people who do care about the university students themselves and they’re not just cash cows for university. I would say definitely this program would have definitely made me see Monash in a more positive light.” (P032, male, domestic student, moderate engagement)  “I think (PEAK) definitely should be something that's done at Monash and is important…everyone should exercise… it's so important for our health… when I exercise, I'm more productive and less stressed and less hard on myself …I just think there should be more done in the space of getting people to feel like exercises are normal and a fun part of life….” (P072, female, domestic, low engagement) |
| The “Kick Off session” PEAK program onboarding was informative, clearly delivered and engaging | | | “It helped me a bit like, you know, being more engaged; just to understand it better. Because when you have that understanding, then you can kind of have a certain expectation as to how you would design your exercise routine.” (P005, female, international student, low engagement) |
| Menu of exercise options | …were generally relevant, acceptable and/or accessible | | “Those [free passes to access the university gym] were instrumental. Without them I don't think I would have gotten three done a week or engaged as much at all.” (P001, female, domestic student, high engagement)  “Even the exercise starter pack that I got like, it was pretty helpful because there are times I don't really want to go to the gym and just work out in my apartment. It just makes it a lot easier” (P005, female, international student, low engagement) |
|  | … but, did not meet some student’s needs or exercise preferences, and/or were difficult to access on the Moodle delivery platform for others | | “If you removed [the online exercise options], it would be fine for me because if I wanted to do this specific exercise I would just go online for it, anyways, and it’s kind of like the same thing anyways. So, it didn’t really make much sense for me to have to come here just [to] find out what videos I should be doing.” (P088, male, international student, low engagement)  “The starter pack, I only use the calendar and the rest I just put in my drawer… I don’t usually use a yoga mat that much. The towel, I don’t use towel that much. Like when I sweat, I just use tissue. Other things, it’s the water bottle, I already have water bottle.” (P115, female, international student, moderate engagement) |
| Educational and motivational videos | …were informative, engaging, relatable to student life and/or prompted reflection for some | | “The “learn a bit” videos, I really enjoyed. I found them engaging and fun. I especially liked the ones where it made me sit and actively think about something like the, what is your why video. The why video was my favourite and then my other favourite was the one when there was cake and Doritos on the bed.” (P001, female, domestic student, high engagement) |
|  | …however, were difficult to access from the Moodle delivery platform, not engaging and/or perceived as irrelevant for some | | “I found the first few [learn a bit videos] not that engaging for me because I've already experienced the benefits of exercise.” (P065, male, international student, moderate engagement)  “I don’t usually watch this type of video that much because I think everyone knows doing some sport is good for your health, so that’s what I think they tell you, or what’s the benefits.” (P115, female, international student, moderate engagement) |
| “PEAK Points” reward system | …was an appealing concept for all | | “when you gather points, they are accumulating and then you can use them to cash in whatever you like, either with sessions or with coffee. Give yourself some kind of small treat because you work hard” (P115, female, international student, moderate engagement) |
|  | … however, not salient, irrelevant and/or difficult to access for most | | “I was thinking that the rewards are kind of not incentivising.” (P088, male, international student, low engagement)  “I just didn't redeem any of [the points]. I know that if you get eight points you got a coffee but I don't drink coffee. If you get 16 points, you get that gym PT session or something, again, I didn't use it because I live so far away. The points are just sitting there.” (P005, female, international student, low engagement)  “I thought the PEAK Points were quite good. It was a very easy system to understand, I think. The only thing I would probably prefer is if the point buying system could be improved, because it was a little bit convoluted where you had to text someone and text them what you want and you get a text back with codes and vouchers and then it’s very difficult to see which codes you’d used already.” (P032, male, domestic student, moderate engagement) |
| WhatsApp social support group | …helped foster a sense of community and solidarity among peers, and/or accountability to the program and exercise for many | | “The PEAK WhatsApp chat was good. I like how everybody like would post pictures or you know, tell about their experience and why they got into PEAK.” (P005, female, international student, low engagement) |
|  | …however, overwhelmed with content, too intimidating to engage with, and/or irrelevant for many | | “I joined [the whatsapp group] initially, but then I didn’t end up having a look at it. I just thought there were too many messages. I think there were quite a few long messages and then I thought I wouldn’t be able to keep up with it, so I didn’t want to look at it.” (P099, male, domestic student, moderate engagement) |
| “PEAK Packs” exercise groups | …were acceptable, and/or provided social accountability among the groups who remained in tact | | “I think [having the option to join a PEAK Pack] is really nice, because its one of the main reasons that I registered [for PEAK] in the first place” (P088, male, international student, low engagement) |
|  | …however, lacked cohesion and group responsiveness for those whose groups disbanded, and/or instilled disconnection from the program for some not involved in a “PEAK Pack” | | “it’s kind of demoralising because you have the group chat with five people for the PEAK pack, and then initially it was all good because we would ask, hey, do you want to go to the session today? It started out like people would respond, but as time went on they started responding less, or not responding at all. It left people hanging, in a sense. It made it harder, or it made it so that you wouldn’t put in the effort to ask them, if that makes sense. You’d ask some people, but not all, sort of thing.” (P088, male, international student, low engagement)  “I remember reaching out to the specific [PEAK Pack] group and it'll take days for people to reply. So, it might be just that because they're busy or maybe PEAK is just not a priority for them. I don't really know…I was like, I hate this group.” (P065, male, international student, moderate engagement)  “I felt like if you weren’t really in a PEAK Pack, you didn’t feel like you belonged to PEAK really, it was more so you were one amongst the many.” (P032, male, domestic student, moderate engagement) |
| Exercise trackers | …were quick and easy to complete, aided accountability and/or served as a reminder to exercise for a minority | | “The exercise trackers were short and they were a lot easier to complete.” (P005, female, international student, low engagement)  “I need to exercise now so I can put it on the exercise tracker, so that motivated me” (P073, female, domestic student, high engagement) |
|  | …however, did not accurately capture the exercise students were engaging in, lacked clarity about what duration or type of exercise could be logged, were easily forgotten, burdensome and/or a low priority for the majority, and demotivating for some students if it highlighted their inactivity | | “There’s an app called 7 Minutes Fitter, which I use a bit. It’s like, is it worth logging that activity or not? Because it usually wants activities that are 10 minutes plus, but if I’ve done that two times in a day, do I log that? Do I not?” (P032, male, domestic student, moderate engagement)  “I missed the SMS and then I just stopped doing [the weekly exercise tracker]” (P038, female, international student, moderate engagement)  “I’d say [the weekly exercise tracker] is a bit demotivating. Because you see all these tracker notifications, like, oh I haven’t exercised in the – it, like, makes you feel a bit worse each time it comes up.” (P088, male, international student, low engagement) |
| Wellbeing trackers took too long to complete and questions lacked clarity | | | “The wellbeing trackers I think were a bit long. Sometimes I wouldn't end up completing them because it clearly said it would take eight to 10 minutes, and I was a bit like, it can be a bit long” (P005, female, international student, low engagement)  “It's the questions about how many times you feel depressed last week? Something like that. Because, actually, I don’t feel depressed, but sometimes – I don’t know the definition of the depressed is, what kind of emotion will be defined as depressed?” (P083, female, international student, low engagement) |
| Program outcome reports | …were informative, motivating and/or rewarding for most | | “I just had a quick flick through the report that you sent out to everyone with the graphs and I loved having that as a final thing that displays how we went. Then I went oh, even though I had some extra bad weeks in there I still came out doing better than I did at the start and that feels really good to look at.” (P001, female, domestic student, high engagement)  “…the fact I’m going to get a report at the end, that was what motivated me to do [the exercise and wellbeing trackers], and to be consistent. I want to produce good results I’ll see how my optimal performance and exercise will change. I want to see that difference.” (P073, female, domestic student, high engagement) |
|  | …however, many were not aware that they would receive an outcome report | | “I didn't really register that I would get a report at the end.” (P014, male, domestic student, high engagement) |
| Moodle digital delivery platform | …was convenient to access for some | | “I had all my uni related things in the same spot and I was used to the Moodle program as well, so that was good to be familiar with the layout of everything, so I liked it” (P073, female, domestic student, high engagement) |
|  | …however, too formal, unappealing, and/or burdensome for most | | “[I] feel like I'm learning something, not the things I can enjoy, so maybe it's a formal platform. It's a little bit formal, not like the things I can relax myself.” (P083, female, international student, low engagement)  “I think maybe – it just felt busy, even though maybe it’s quite a quick thing to do, you just have to put effort in or log into Moodle. I feel like if it was an app on the phone – I probably did have enough time to look on Moodle to watch the two-minute videos. That doesn’t take too long compared to spending time on social media or messaging on the phone, which you might do quite a bit. So, if it was on the phone and I got a notification I might do it on the way walking or just while commuting, for example. On Moodle you have to do it on your laptop, so it’s a bit harder than mobile devices.” (P099, male, domestic student, moderate engagement) |
| Cultural considerations | The cultural inclusivity of the exercise options could be enhanced | | “As an international student I feel like - well, you know, I'm fine because I'm a nursing student and I have some knowledge about the terminology, but for the student who has language barriers, probably I think when providing the exercise in the other language. Sometimes they - if they can't understand that it might take some time for them to try to translate or understand in their brain, so that may be a little bit hard to really put themselves into the exercise because they need to be understanding the video.” (P058, female, international student, low engagement)  “I'm from Chinese background, so there are lots of different kinds of exercise app in my country, I think. So there's one called Keep… it actually uses Chinese to guide you…that’s also the reason why I choose a Chinese app. I just want to make myself feel comfortable.” (P058, female, international student, low engagement) |
| Mechanisms of behaviour change | | | |
| Psychological Capability | Increased knowledge about the variety of types of exercises that can be incorporated into an exercise regime | | “Also, with some of the exercise videos that was - because a lot of these exercises were things that I wouldn't usually do, just having that knowledge like, this is something I can do and incorporate further into my exercise routine, that was also good.” (P005, female, international student, low engagement) |
|  | Increased knowledge about how exercise is beneficial | Increased knowledge about how exercise can improve mental, cognitive and brain health | “I remember learning a bit more about the effects that exercise has on a few different things. They talked about memory in the videos and just mood in general. So, I think I just – it strengthened my understanding of how exercise has beyond physical benefits.” (P099, male, domestic student, moderate engagement)  “I learned about how [exercise] actually helps with the brain, with the brain function and release your dopamine and help your mood, help relieve your stress. Which I didn’t think about before, [unclear] exercise or to choose some knowledge about how it actually helps your body, the benefits.” (P115, female, international student, moderate engagement) |
|  |  | Increased knowledge about the long-term benefits of consistently engaging in exercise | “Yes [I learnt something new from PEAK]. Consistency is best and benefits are reaped over time/” (P065, male, international student, moderate engagement) |
|  | Learning from student peers, especially those who were more experienced exercisers, and the PEAK program team, especially the exercise physiologist | | “I liked how I could ask any questions [in the whatsapp group] and get some advice from everyone and hearing about everyone’s journeys, I found that really interesting.” (P073, female, domestic student, high engagement)  “In the gym, it’s very valuable to be able to talk to people who know much more about gymming and to get their advice on things.” (P104, male, domestic student, moderate engagement)  “Yes, [I gained new knowledge from PEAK] through [the PEAK exercise physiologist] and the PT session…I asked [the PEAK exercise physiologist] a range of things…He talked about how sit-ups are perhaps not the best form of ab exercise and he gave good advice on that. I asked him about how strength training versus cardio will influence brain – how active you feel and how much energy you have, so he talked a bit about that. That was interesting to hear” (P104, male, domestic student, moderate engagement) |
| Physical Opportunity | Easy access to a variety of exercise options (online, on-campus and at home) enabled some students to engage in a wider variety of exercises | | “Previously I don’t exercise, I do yoga and through PEAK I know a new type of exercise we can do, or have a lot of options you can choose, cardio, strength…” (P115, female, international student, moderate engagement)  “I like how they had just a range of options. Even within the exercises that they suggested, there were just so many, so many you could choose from. Even how you wanted to do them via video, via an app, it was quite helpful.” (P005, female, international student, low engagement) |
|  | Free exercise options, including free passes to the gym, increased the financial accessibility of exercise | | “Because I’m not able to afford a gym membership for myself so I found the passes really helpful for me because I could take the opportunity to go to a gym” (P073, female, domestic student, high engagement) |
|  | Prompts and cues via the Moodle platform, reminder text message to complete the exercise tracker, and/or seeing items from the exercise starter kit reminded students to exercise | | “I will open Moodle every day, because when I see the home of Moodle, I will see the PEAK program, and maybe I will remind myself to – it's time to do some exercise now.” (P083, female, international student, low engagement)  “I liked having the tote bag it was kind of a reminder that I need to exercise.” (P072, female, domestic, low engagement) |
| Social Opportunity | Increased opportunities to connect with others | Connecting with peers increased accountability, motivation and enjoyment of exercise | “I used to be the kind of person that's like, I want to [exercise] alone… But through this program, I figured out that exercising with people is actually very fun, and it can help actually help motivate you to go further, above and beyond. Normally I would be tired at this point, but because there's people around me, I’m just going to work a little bit harder. So, yes, it did motivate me to work out socially. (P065, male, international student, moderate engagement) |
|  |  | Forming and strengthening friendships, especially for the purpose of exercising together | “I want to do exercise and I want to make more friends. Making more friends is an easy thing to do [through] more exercise but I didn’t think of how making friends who do exercise contributes to exercise. That’s new to me.” (P104, male, domestic student, moderate engagement) |
|  |  | Being part of a likeminded exercise community encourages exercise | “One thing that really helped me go through, besides the tracker and all the sciencey and stuff and all the benefits of exercise, is the community. Having just like-minded people working towards the same goal and knowing that - because usually in university, if you want to join a sports group you have to have a certain fitness level and if not, you're discouraged. Otherwise, if you don't really have a friend group maybe that have similar interests as you and you don't really want to initiate that conversation. But with PEAK because you facilitate everything, it just makes it a lot easier.” (P065, male, international student, moderate engagement) |
| Automatic Motivation | Enjoyment of exercise increased | | “…exercise changed from being an obligation to something that I actually enjoy.” (P065, male, international student, moderate engagement) |
|  | Ability to establish a consistent exercise routine improved | | “I honestly have been putting this PEAK experience up there in the life changing experiences that I've had. The fact that for the first time ever that I remember I am consistently exercising three times a week. I am doing that self-motivated now because the gym pass has run out and I used to be exercising with my housemates, I'm doing that less now, but I am still going out there and I am exercising three times a week. That's never happened before…So just the [exercise] consistency and me knowing that I can commit to consistency makes me feel really good too and PEAK has had a big part of that so it makes me feel really good.” (P001, female, domestic student, high engagement) |
|  | Experiencing the health benefits of exercise was reinforcing | Experiencing the mental health benefits of exercise | “I had quite a stressful semester with both uni and family stuff… once I started exercising and getting a routine it was very clear that I felt much better the days that I did exercise than the days I didn't. That it was providing mental clarity, better emotional regulation and mood, et cetera. So it just made me feel so good that I was motivated to exercise so that I could feel good again.” (P001, female, domestic student, high engagement)  “The best comparison I can make is between this semester and last semester, because last semester I barely did any exercise at all really… I completely burnt out and couldn't really do anything… main thing that regular exercise has given me [this semester] is resilience to those sorts of times and motivation and energy to not procrastinate as much...” (P014, male, domestic student, high engagement) |
|  |  | Experiencing the cognitive health benefits of exercise | “whenever I exercised, especially in the morning, I'm able to concentrate on my work better, and I'm able to have a better flow of a day and have something that clears my mind” (P038, female, international student, moderate engagement) |
|  |  | Experiencing the physical health benefits of exercise | “PEAK was a fantastic opportunity to get points and do this thing with friends and make it so fun and also get fit again.” (P001, female, domestic student, high engagement) |
|  | Feeling intrinsically motivated to exercise instead of extrinsically motivated | | “…before I would think the purpose of doing exercise is to lose weight… but now I maybe interested for to let me, kind of, enjoy the exercise, and I could actually…feel wellbeing of my mental state, and I will feel quite relaxed after I finished exercise” (P083, female, international student, low engagement) |
| Reflective Motivation | Motivation to exercise | The credibility of the program content and team was motivating | “Just knowing that the people behind PEAK are such qualified individuals was a very big motivation factor for me and meeting them” (P113, male, international student, moderate engagement) |
|  |  | Consistent messaging about how exercise can support students to study well, and the mental, cognitive and brain benefits of exercise was motivating | “first semester I was really focused on just study that I completely lost exercise. I have heard that exercise does help but it was not enforced on me… having a program that talks about it regularly really motivated me” (P073, female, domestic student, high engagement) |
|  | Rewards to exercise | Receiving “PEAK Points” and rewards increased motivation to exercise | “I love points. In the first few weeks when I was - before my habits were sticking, I didn't care about the exercise, I didn't care about the mental capacity that I gained or the feelings, I just wanted points.” (P001, female, domestic student, high engagement) |
|  | The goal of exercising three times a week was helpful | | “I think what PEAK helped was maybe initiate me to exercise because I thought about trying to hit that target of three times a week.” (P099, male, domestic student, moderate engagement) |
|  | The program goals and content was appropriately tailored to students aspirations, lifestyle, and their barriers and facilitators to exercise | | “I really like this goal of improving mind mood and marks, I find that nice to hear as a student because you want to improve those things especially being full time and all the stress, so I really like that initiative.” (P073, female, domestic student, high engagement)  “It seems [the program] know where the low points, where's the high points. I think you’ve put the sciencey bit during that part where students are still motivated, so they don't need that much motivation to keep going. Then towards the end of the semester, students are down here, right? So, your videos are more motivational style. That was good. I think it was very relevant.” (P065, male, international student, moderate engagement) |
